# Differential coding of uncertain reward in rat insular and orbitofrontal cortex

**DOI:** 10.1038/srep24085

**Published:** 2016-04-07

**Authors:** Suhyun Jo, Min Whan Jung

**Affiliations:** 1 Center for Synaptic Brain Dysfunctions, Institute for Basic Science, Daejeon 305-701, Korea; 2 Neuroscience Graduate Program, Ajou University School of Medicine, Suwon 443-721, Korea; 3 Department of Biological Sciences, Korea Advanced Institute of Science and Technology, Daejeon 305-701, Korea

## Abstract

Anterior insular and orbitofrontal cortex (AIC and OFC, respectively) are known to play important roles in decision making under risk. However, risk-related AIC neural activity has not been investigated and it is controversial whether the rodent OFC conveys genuine risk signals. To address these issues, we examined AIC and OFC neuronal activity in rats responding to five distinct auditory cues predicting water reward with different probabilities. Both structures conveyed significant neural signals for reward, value and risk, with value and risk signals conjunctively coded. However, value signals were stronger and appeared earlier in the OFC, and many risk-coding OFC neurons responded only to the cue predicting certain (100%) reward. Also, AIC neurons tended to increase their activity for a prolonged time following a negative outcome and according to previously expected value. These results show that both the AIC and OFC convey neural signals related to reward uncertainty, but in different ways. The OFC might play an important role in encoding certain reward-biased, risk-modulated subjective value, whereas the AIC might convey prolonged negative outcome and disappointment signals.

Humans and animals often choose a safe over a risky option even when the safe option has a lower expected value, indicating that subjective value of an expected outcome is affected by risk^1^,^2^,^3^. Neural systems associated with decision making under risk are not completely understood, but studies so far suggest anterior insular cortex (AIC) and orbitofrontal cortex (OFC) as two key structures in this process. Numerous brain imaging studies have found activation of the AIC^4^,^5^,^6^,^7^,^8^,^9^,^10^ and OFC^4^,^11^,^12^ in association with risk. Lesion studies in humans^11^,^13^,^14^ and animals^15^,^16^,^17^,^18^ have also found altered choice behavior during decision making under risk following lesions to the AIC or OFC. In studies comparing AIC and OFC lesion effects in the same behavioral task, distinct lesion effects were observed, suggesting distinct roles of the AIC and OFC in risky decision making. In humans, damages in the OFC were associated with increased betting regardless of the odds of winning, whereas patients with damages in the AIC failed to adjust their bets by the odds of winning^13^. In rats, OFC and AIC inactivation increased and decreased risk preference, respectively^16^. Lesion effects are not consistent across studies, however. For example, contrary to the above finding^16^, OFC lesions promoted preference for certain reward in rats^15^,^17^.

Compared to brain imaging and lesion studies, progress has been relatively slow in revealing neural correlates of risky decision making in the AIC and OFC. Single neuron recording studies have found risk-coding neurons in the monkey OFC^19^,^20^. However, in the rat OFC, neural correlates of risk have not been clearly demonstrated. OFC neurons in rats have been found to encode ‘decision confidence’ before choice outcome was revealed^21^ and to show differential responses to large uncertain vs. small certain rewards in risk-preferring, but not in risk-neutral animals^22^. However, few OFC neurons signaled risk information in rats responding to olfactory cues predicting reward with different probabilities^23^, and OFC neural activity seemingly responsive to risk has been proposed to represent ‘acquired salience^24^’ rather than risk^25^. In the AIC, there has been no neurophysiological study investigating risk-related neural activity so far. Thus, although there exists a substantial body of evidence for the involvement of the AIC and OFC in decision making under risk, their specific roles and the nature of information processed in each brain structure are unclear.

The following two issues are therefore important to understand how the AIC and OFC contribute to decision making under risk: 1) determining whether and how OFC neurons in rats convey neural signals related to risk, and 2) examining neuronal activity related to risk in the AIC. Further, it would be desirable to compare AIC and OFC neural activity in the same subject in the same behavioral paradigm. In the present study, to address these issues, we compared single unit activity in the AIC and OFC in rats responding to five distinct cues predicting reward with different probabilities. We found both similarities and differences in neural signals related to uncertain reward between the AIC and OFC, which might underlie their distinct contributions to decision making under risk.

## Results

### Behavior

**Figure 1:**
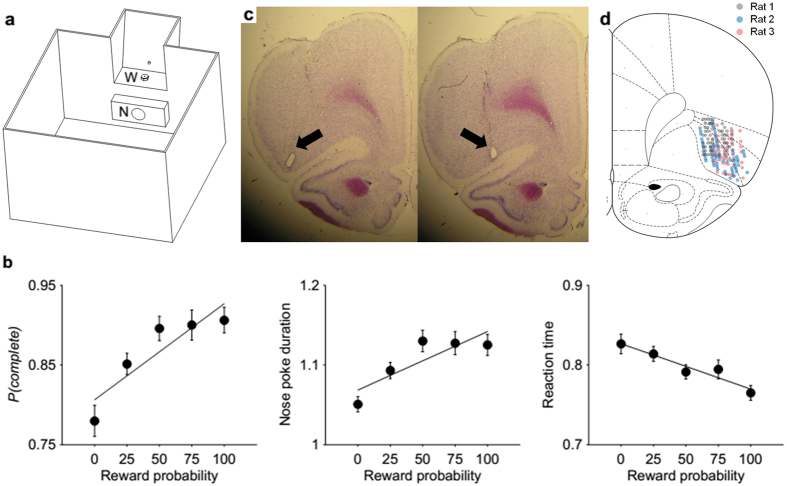
Animal behavior and recording sites. (**a**) Behavioral apparatus. N, nose poke hole; W, water-delivery nozzle. One of five different auditory cues, signaling five different reward probabilities (0, 25, 50, 75 and 100%), was delivered for 1 s during the animal’s nose poke (minimum 1 s to complete a trial). A blue LED (the small circle above the water-delivery nozzle) was turned on at nose poke exit, and the animal’s arrival at the reward port triggered probabilistic water delivery. (**b**) Behavioral performance. The proportion of complete trials (left), nose poke duration (middle), and reaction time (the time interval between nose poke exit and water-port entry; right) as a function of reward probability. The lines were determined with linear regression analyses. Error bars, SEM. (**c**) Single units were recorded from the AIC and lateral OFC. The photomicrographs are coronal sections of the rat brain showing marking lesions (arrows) in the AIC (left) and OFC (right). (**d**) The diagram is a coronal section view of the rat brain at 3.2 mm anterior to bregma. Each circle represents one recording site that was determined based on histology and electrode advancement history. Different colors represent recordings from different rats. One to five units were recorded simultaneously from each site. Modified with permission from Paxinos and Watson, 1998.

Three rats were trained in a probabilistic Pavlovian appetitive conditioning task in an open chamber (Fig. 1a). The animal’s nose poke following trial onset (signaled by a blue LED inside the nose poke hole) triggered one of five auditory tones that lasted for 1 s. The five different auditory tones (1.2, 2, 5, 9 and 14 KHz) were associated with five different reward probabilities (0, 25, 50, 75 and 100%) with cue-reward probability combinations varied across rats. The animals were required to maintain nose poke ≥1 s (i.e., until the offset of the auditory cue). A blue LED at the water port was turned on at the time of nose poke exit, and the animal’s arrival at the water port triggered water delivery (30 μl) with a given probability. In incomplete trials (nose poke duration <1 s), the blue LED at the water port was not turned on and a 5~10 s inter-trial interval was introduced. The animals had to approach to the water port in all trials (including when the cue predicted 0% reward probability) in order to proceed to the next trial.

The animals completed the majority of trials (maintaining nose poke ≥1 s) during unit recording sessions (rat A, 93.0 ± 2.9; rat B, 72.7 ± 6.2; rat C, 89.6 ± 4.2%; mean ± SD). The proportion of trial completion tended to increase as a function of reward probability and, conversely, reaction time (the time interval between nose poke exit and water-port entry) tended to decrease with the reward probability (Fig. 1b, left and right, respectively; linear regression analysis, *p* = 0.039 and *p* = 0.010, respectively). These results provide evidence that the animals formed cue-reward probability associations. Nose poke duration did not show significant relationship with the reward probability, however (Fig. 1b, middle; *p* = 0.066). Examining behavior of individual rats, we found both positive (two rats) and negative (one rat) relationships between nose poke duration and reward probability. The behavioral strategy dealing with the trade-off between minimizing reward delay and avoiding incomplete trial might differ across animals. Some might prefer to wait longer to avoid incomplete trials (at the expense of reward delay) in high reward probability trials, while others use the opposite strategy.

The animals showed stereotypical behavior during nose poke, approach to reward port, and the early phase (~1 s) of the reward period regardless of previous trial outcome. Nose poke duration and reaction time were independent of the previous trial outcome (nose poke duration, after reward, 1.11 ± 0.06 s; after no reward 1.11 ± 0.07 s; mean ± SD; *t*-test, *p* = 0.267; reaction time, after reward, 0.80 ± 0.06 s; after no reward, 0.79 ± 0.06 s; *p* = 0.139). The duration of the reward period differed between rewarded and unrewarded trials (10.27 ± 1.86 and 1.70 ± 0.52 s, respectively; mean ± SD), but it was independent of the reward probability (rewarded trials, one-way ANOVA, *p* = 0.643; unrewarded trials, *p* = 0.608).

### Time courses of value, risk and reward signals

We recorded 156 and 201 putative single units from the AIC and lateral OFC, respectively, of the three rats (the number of recorded units in each rat, AIC, 48, 43 and 65; OFC, 68, 70 and 63; Fig. 1c,d). All units were included in the analysis, but similar results were obtained when only putative pyramidal cells (AIC, n = 126; OFC, n = 154) were included in the analysis (Fig. S1). Similar results were also obtained when those neurons whose estimated recording locations were close to the border between the AIC and lateral OFC were excluded from the analysis (Fig. S2). We first examined neural signals related to reward, value, and risk with a multiple regression analysis (eq. 1) using only complete trials. In the present study, ‘reward’ was the binary trial outcome (delivery of water or its omission; represented as 1 and −1 with a dummy variable), ‘value’ was the reward probability associated with each auditory cue (i.e., objective value; represented as 0, 1, 2, 3 and 4 for 0, 25, 50, 75 and 100% reward probabilities) unless noted as ‘subjective value’, and ‘risk’ was the standard deviation of the trial outcome (represented as 0, 0.433, 0.5, 0.433 and 0 for 0, 25, 50, 75 and 100% reward probabilities; see Materials and Methods). We included reward, value, and risk of the current (trial lag = 0) as well as two previous trials (trial lags = 1 and 2) as independent variables in the regression model in order to examine how long these variables influence AIC and OFC neuronal activity.

**Figure 2:**
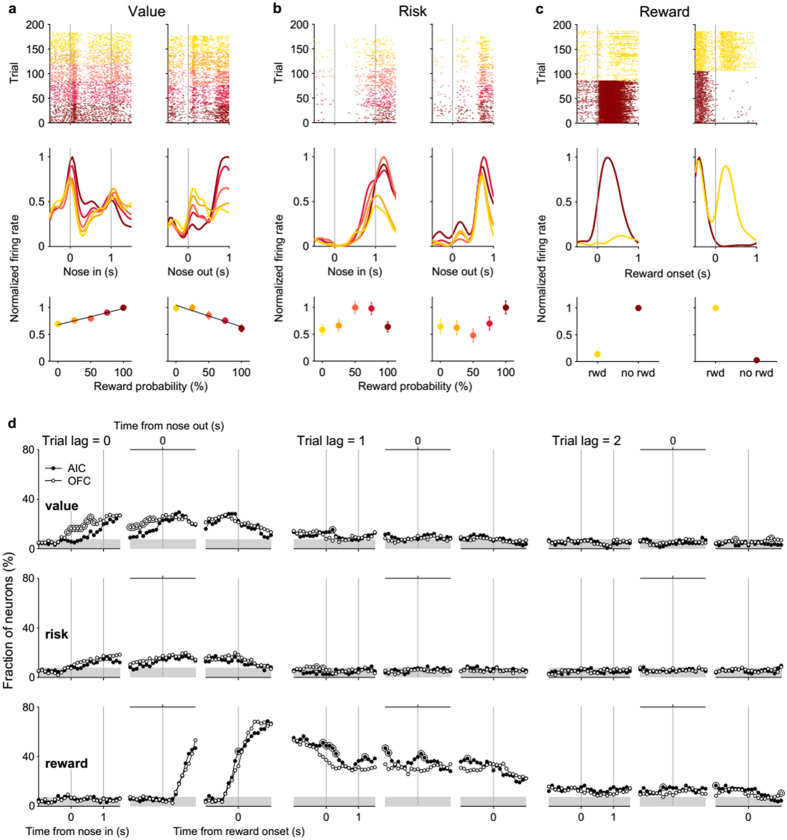
Neural signals for value, risk, and reward in the AIC and OFC. (**a**) Two example neurons significantly responding to current value during the cue (left, OFC neuron) or response period (right, AIC neuron). Time 0 indicates the onset of the cue (‘Nose in’, left) or response period (‘Nose out’, right). Top, spike raster plots. Trial-by-trial neural activity is shown. Each row is one trial and each dot represents one spike. Trials were grouped according to value (i.e., reward probability). Middle, spike density functions. Neural activity was averaged across same-value trials after applying a Gaussian kernel (σ = 100 ms) to each spike. Darker colors indicate higher values. Bottom, mean (±SEM across trials) discharge rate during the cue (left) or response period (right) is plotted as a function of reward probability. (**b**) Two example neurons significantly responding to current risk during the cue (left, OFC neuron) or response period (right, AIC neuron). (**c**) Two example neurons significantly responding to current reward during the reward period (left, OFC neuron; right, AIC neuron). Time 0 indicates reward period onset. (**d**) Temporal profiles of neural signals for value, risk and reward. Shown are fractions of AIC and OFC neurons significantly responsive to value (reward probability), risk (standard deviation of the trial outcome) and reward (trial outcome) in the current (trial lag = 0) and two previous trials (trial lags = 1 and 2) in a 1-s analysis time window advanced in 0.1-s time steps across different behavioral periods (cue, response and reward periods). Vertical lines mark beginning (time 0) or end of a behavioral stage. Shading indicates mean chance level (binomial test, α = 0.05) for the AIC (7.7%) and OFC (7.0%). Large open circles indicate significantly different fractions (χ^2^-test, *p* < 0.05) between the AIC and OFC.

Both the AIC and OFC contained neurons responsive to reward, value, and/or risk. Significant fractions of neurons were responsive to value during the cue (n = 19, 12.2%) and response (n = 39, 25.0%) periods in the AIC as well as in the OFC (cue period, n = 47, 23.4%; response period, n = 45, 22.4%; Table 1; examples are shown in Fig. 2a). Significant fractions of neurons were also responsive to risk in the AIC (cue period, n = 14, 9.0%; response period, n = 20, 12.8%) and OFC (cue period, n = 27, 13.4%; response period, n = 27, 13.4%; Table 1; examples in Fig. 2b). During the first 1 s of reward period, significant fractions of neurons were responsive to reward (i.e., trial outcome) in both brain areas (AIC, n = 92, 59.0%; OFC, n = 135, 67.2%; examples in Fig. 2c).

Figure 2d shows temporal profiles of these neural signals in terms of the fraction of neurons significantly responsive to a given variable in a 1-s time window (similar results were obtained with the analysis using a 0.5-s window) advanced in 0.1-s time steps in reference to nose poke entry (cue period), nose poke exit (response period) and reward port entry (reward period). Both value and risk signals increased following nose poke entry and were maintained above chance level (binomial test, *p* < 0.05) until the reward period in both brain areas. Risk signals returned to chance level within ~1 s following reward port entry, but value signals were maintained, albeit weakly, until the next trial (trial lag = 1). Reward signals arose rapidly at the reward period onset (~70% of neurons were responsive to reward) and stayed above chance level for the next two trials (Fig. 2d). These results show that both the AIC and OFC conveyed significant neural signals for value, risk and reward. However, quantitative differences were found in value and reward signals between the AIC and OFC. First, current (trial lag = 0) value signals were stronger (χ^2^-test, *p* < 0.05) and elevated earlier in the OFC during the cue period (Fig. 2d, top). Second, previous (trial lag = 1) reward signals were somewhat stronger in the AIC (Fig. 2d, bottom; cue and response periods).

### Characteristics of value signals

**Figure 3:**
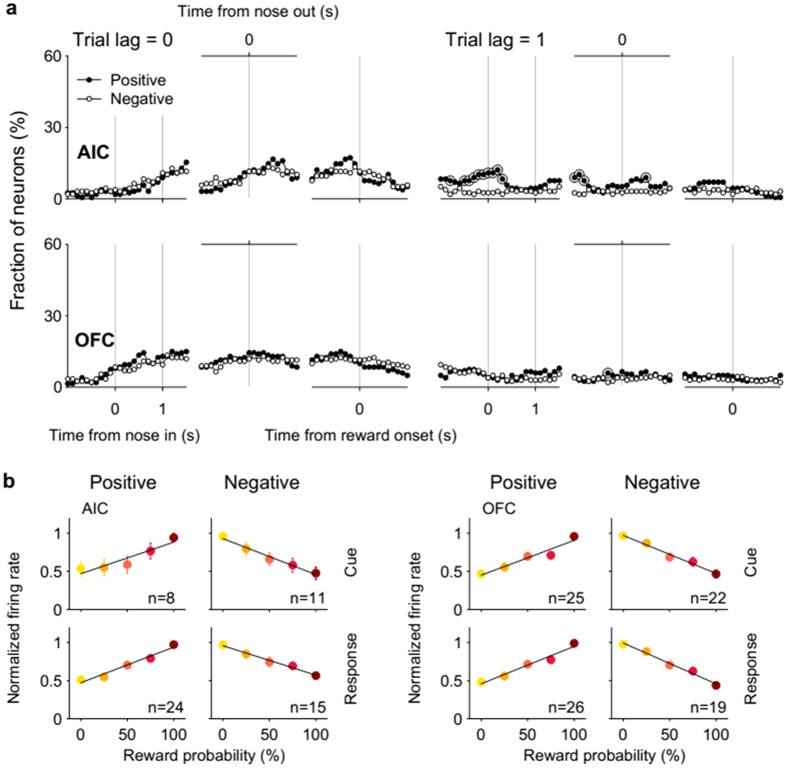
Characteristics of value-responsive neural activity. (**a**) Same plots as in Fig. 2d except that value-responsive neurons were divided into activity-increasing (‘Positive’) and -decreasing (‘Negative’) types and plotted separately. Large open circles indicate significantly different fractions (χ^2^-test, *p* < 0.05) between activity-increasing and -decreasing types. (**b**) Population responses of value-responsive neurons. Mean discharge rates normalized to the peak firing rate of all value-responsive AIC and OFC neurons during the cue and response periods are plotted separately for activity-increasing (‘Positive’) and -decreasing (‘Negative’) types as a function of reward probability. The number in each plot indicates the number of neurons in each category and error bars denote SEM across neurons. Each line is an outcome of linear regression.

For further characterization, we examined neuronal responses to value, risk and reward according to their response polarity. For the current (trial lag = 0) value-coding neurons, few time windows contained significantly different numbers (χ^2^-test, *p* < 0.05) of activity-increasing vs. activity-decreasing neurons as a function of value in both regions (Fig. 3a; population responses shown in Fig. 3b). However, of the previous (trial lag = 1) value-coding neurons, significantly larger numbers increased than decreased their firing rates according to previous value around nose poke entry in the AIC (an example shown in Fig. S3), which was not the case in the OFC (Fig. 3a). These results indicate that AIC neurons tended to increase their activity as a function of previous value at the time of nose poke entry.

### Characteristics of risk signals

**Figure 4:**
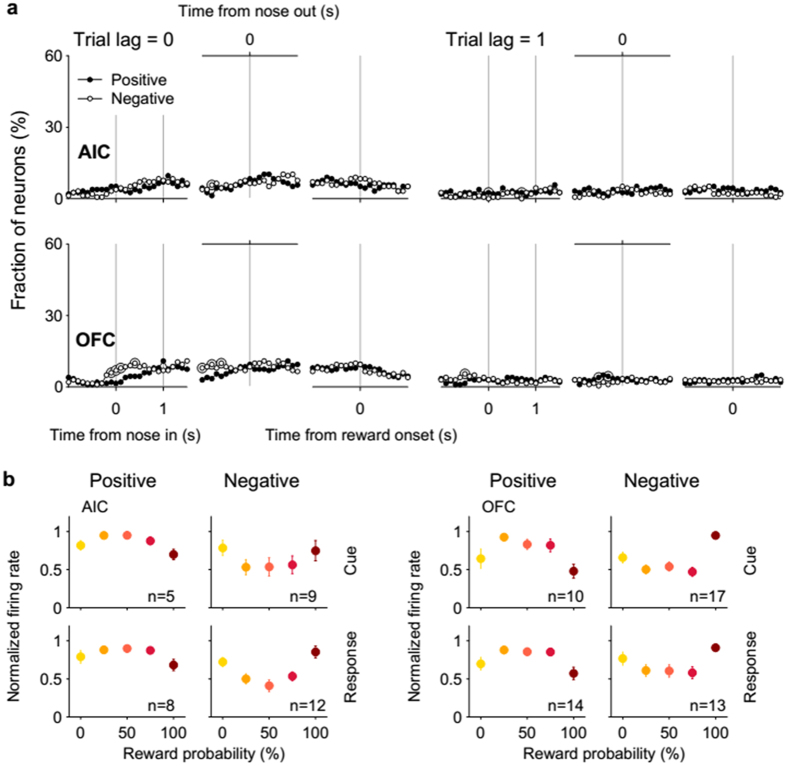
Characteristics of risk-responsive neural activity. Same plots as in Fig. 3 except that risk-responsive neurons, instead of value-responsive neurons, were analyzed. (**a**) Risk-responsive neurons were divided into activity-increasing and-decreasing types and plotted separately. (**b**) Population responses of risk-responsive neurons during the cue and response periods plotted separately for activity-increasing and -decreasing types.

**Figure 5:**
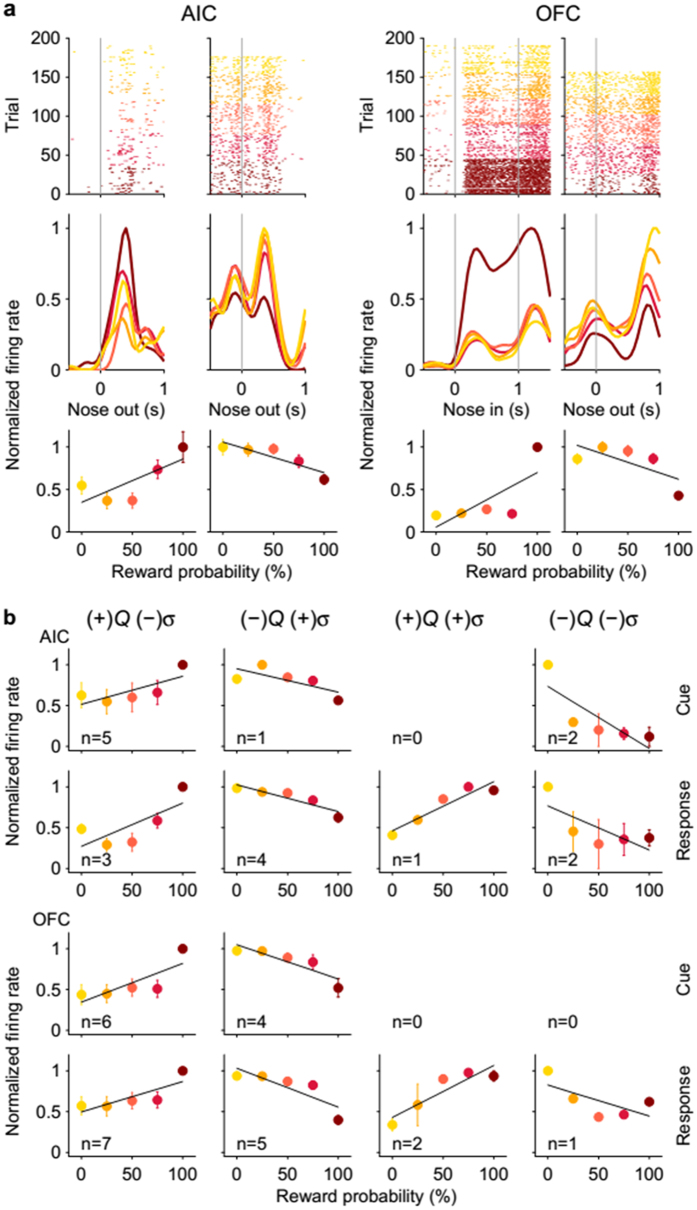
Neural responses concurrently coding value and risk. (**a**) Four example neurons concurrently coding value and risk during the cue or response period. Same format as in Fig. 2a. (**b**) Population responses of all AIC and OFC neurons concurrently coding value and risk during the cue and response periods. The neurons were divided into four groups depending on their response directions (+ or −) to value (*Q*) and risk (*σ*). Same format as in Fig. 3b.

Of all risk-coding neurons, similar numbers showed increased and decreased responses to risk in the majority of analysis time windows in the AIC. However, in the OFC, more neurons (χ^2^-test, *p* < 0.05) decreased than increased their activity as a function of risk during the early cue period (trial lag = 0, Fig. 4a; population responses shown in Fig. 4b). Because both value and risk signals were significant in the AIC and OFC, we examined whether these signals tended to be coded jointly by individual neurons. Value-coding neurons tended to code risk as well more than expected by chance during the cue period in the AIC (*p* = 2.1×10^−5^; OFC neurons showed only a trend, *p* = 0.072) and during the response and reward periods in both areas (*p* values < 0.01; Table 1, top), indicating conjunctive coding of value and risk signals in AIC and OFC neural populations (examples and population responses of neurons concurrently coding value and risk are shown in Fig. 5). Note that those neurons encoding both value and risk passed two independent statistical tests; a neuron to be significantly responsive to both risk and value purely by chance is only 0.25% (one out of 400).

On visual examination, a substantial portion of risk-responsive OFC neurons responded only to the cue predicting 100% reward delivery (the third example in Fig. 5a). To assess the proportion of risk-responsive neurons specifically responding to certain reward, we compared how well two different regression models [base model (eq. 1) vs. certain reward model (eq. 2)] explained activity of risk-responsive neurons. Responses of the majority of risk-responsive OFC neurons were better explained (in terms of Bayesian information criteria) by the certain reward model (eq. 2) than the base model (eq. 1) during the cue period (23 out of 27, 85.2%; comparison with chance level (50%), χ^2^-test, *p* = 2.6×10^−4^), but not during the response period (16 out of 27, 59.3%, *p* = 0.336). There was no tendency for either model to explain AIC neural responses better than the other model during the cue (8 out of 14 better explained by the certain reward model, 57.1%, *p* = 0.593) or response period (11 out of 20, 55.0%; *p* = 0.655). These results show that OFC neurons preferentially coded certain reward rather than graded risk signals during the cue period. As expected, of the certain reward-coding neurons during the early (first 0.5 s) cue period (i.e., risk-responsive neurons whose activity were better explained by the certain reward model than the base model; 15 out of 20 risk-responsive neurons), the majority had negative coefficients for the risk term (i.e., they elevated rather than reduced their firing rates in response to the certain reward-predicting cue; 14 out of 15, 93.3%; comparison with chance level (50%), χ^2^-test, *p* = 7.9×10^−4^), which explains why more OFC neurons decreased than increased their activity as a function of risk during the early cue period (Fig. 4a).

Because OFC neural responses to reward uncertainty have been proposed to be better explained by ‘acquired salience’, which is the weighted sum of cue-outcome and cue-no-outcome associative^24^, rather than risk^25^, we examined the possibility that seemingly risk-responsive neurons actually responded to acquired salience. For this, we compared how well neuronal activity during the cue period is explained by the acquired salience (eq. 3) vs. risk (eq. 4) models (see Materials and Methods). Of all analyzed neurons, the difference in variance explained by the risk and acquired salience models (variance explained by risk model–variance explained by acquired salience model) was significantly larger than zero in the OFC (*p* = 0.009, Wilcoxon-signed rank test), but not in the AIC (*p* = 0.951; Fig. S4a). Thus, OFC neuronal activity was better accounted for by risk than acquired salience, and we failed to obtain evidence favoring the acquired salience model in the AIC. Similar results were obtained with a sliding window analysis (Fig. S4b).

### Characteristics of reward signals

**Figure 6:**
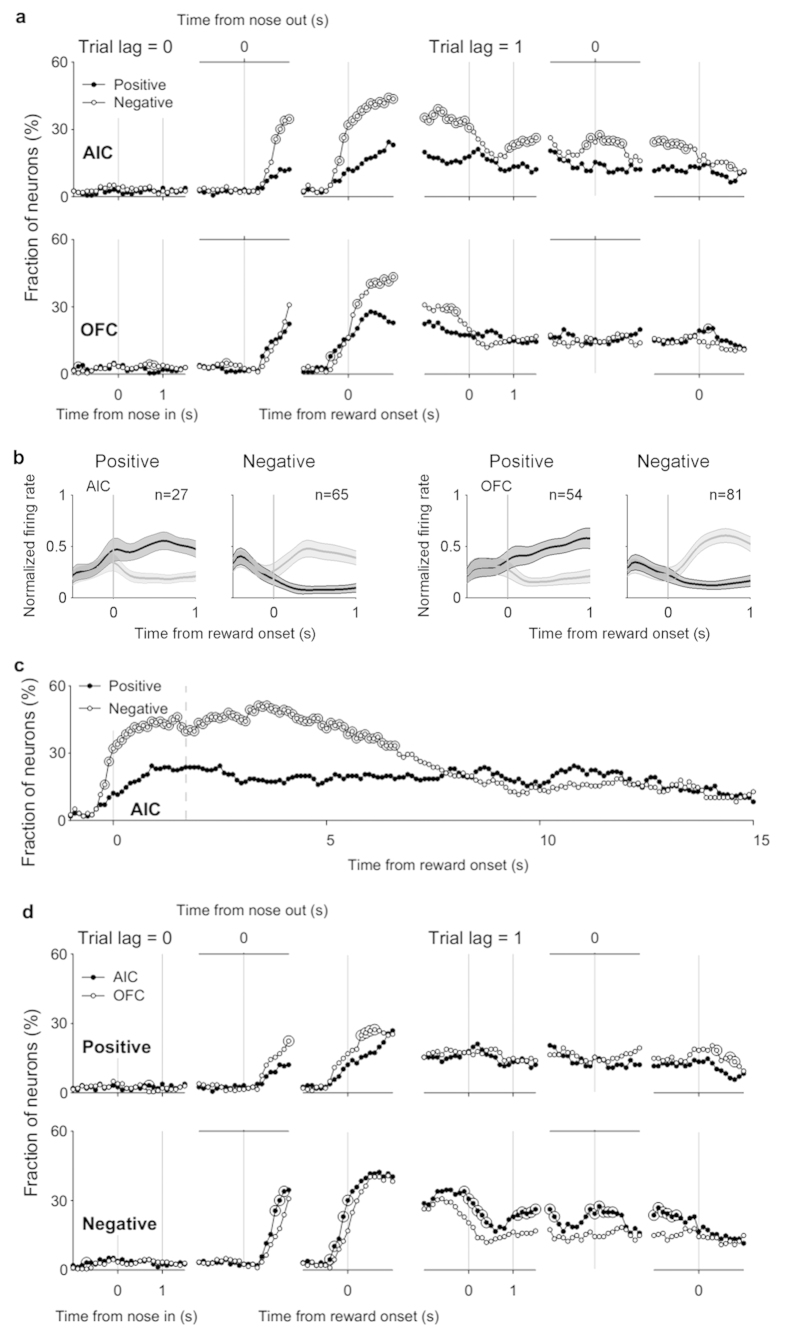
Characteristics of reward-responsive neural activity. (**a**) Reward-responsive neurons were divided into activity-increasing and -decreasing types and plotted separately. Same format as in Fig. 3a. (**b**) Population responses of reward-responsive neurons. Mean peak-normalized discharge rates of all reward-responsive AIC and OFC neurons during the first 1 s of the reward period are plotted separately for activity-increasing and -decreasing types. Dark and light tones represent rewarded and unrewarded trials, respectively. Shading, 95% confidence interval. (**c**) Positive and negative reward signals in the AIC were examined for a prolonged time period aligning trials to the reward period onset. The dashed vertical line denotes the mean duration of unrewarded trials (1.704 s). (**d**) Positive and negative reward signals were compared between the AIC and OFC. Fractions of reward-responsive neurons are shown separately for activity-increasing (‘Positive’) and -decreasing (‘Negative’) types.

Reward-responsive neurons were significantly more likely to decrease than increase their firing rates when reward was delivered in both brain areas (χ^2^-test, *p* < 0.05) (Fig. 6a; population responses shown in Fig. 6b). This difference disappeared by the onset of the next cue period in the OFC, but persisted until the next reward period in the AIC (example AIC neurons coding previous reward are shown in Fig. S5). When we examined reward signals of the AIC for 15 s since the reward period onset, more AIC neurons decreased than increased their firing rates for a prolonged time (>5 s) after reward delivery (Fig. 6c). Although the animal’s behavior in the later phase of the plot [after ~1.7 s (mean reward period duration in unrewarded trials) following reward period onset] differed between rewarded and unrewarded trials, there is no *a priori* reason to expect AIC neurons to show higher, rather than lower, neural activity following negative outcome because of behavioral variations. It would be highly unlikely to see consistently higher neural activity following negative outcome across multiple analysis time windows (>5 s) because of behavioral variations.

Comparing between the AIC and OFC, the fraction of neurons that significantly decreased their activity to previous reward delivery was significantly larger in the AIC than OFC during the cue and response periods (trial lag = 1, Fig. 6d). Conversely, the fraction that significantly increased activity was significantly smaller in the AIC than OFC during the reward period (trial lag = 0, Fig. 6d). Thus, AIC neurons showed a strong tendency to increase their firing rates in response to negative outcome (reward omission) that persisted until the next trial.

**Figure 7:**
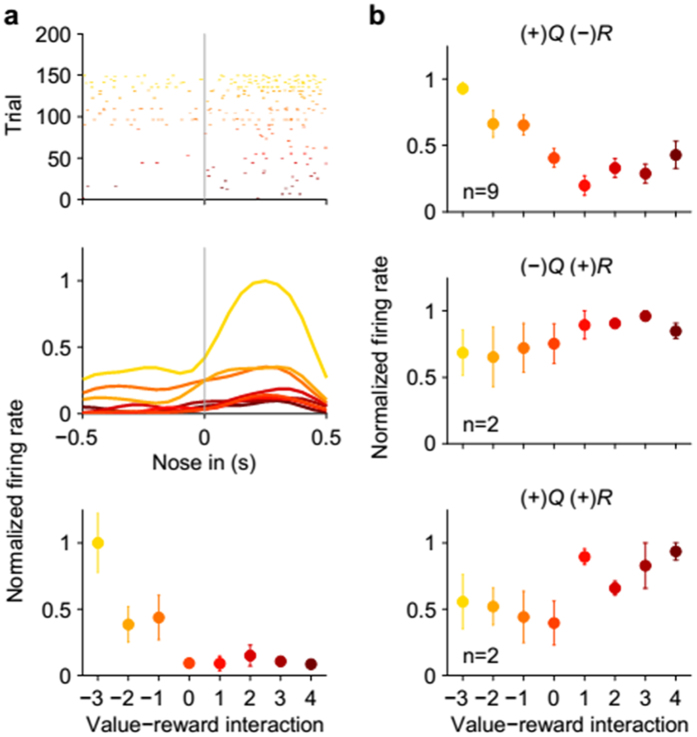
Activity of AIC neurons concurrently coding previous value and previous reward. (**a**) An AIC example neuron concurrently coding value and reward in the previous trial during the 1-s time window centered around cue period onset. Top, a spike raster plot; Middle, spike density functions (σ = 100 ms); Bottom, mean (±SEM across trials) discharge rates during the 1-s time window. Trials were grouped according to value-reward interaction (value, 0, 1, 2, 3 and 4 for 0, 25, 50, 75 and 100% reward probability, respectively; reward, −1 and 1 for unrewarded and rewarded trials, respectively). (**b**) Mean (±SEM across neurons) peak normalized firing rates of all AIC neurons concurrently coding value and reward in the previous trial during the 1-s time window centered around cue period onset. The neurons were divided into four groups depending on their response directions (+ or −) to value (*Q*) and reward (*R*). No neuron belonged to the (−)*Q*(−)*R* group.

That AIC neurons tended to elevate their activity after negative outcome (Fig. 6a; examples shown in Fig. S5) and as a function of previous value (Fig. 3a; examples shown in Fig. S3) raises the possibility that some AIC neurons might represent negative prediction error signals. Consistent with this possibility, during the 1-s time window centered around the nose poke entry, previous reward-coding neurons were significantly more likely to code previous value as well than expected by chance (*p* = 0.029, Table 1, bottom), indicating conjunctive coding of previous reward and previous value signals at the time of nose poke entry (an example and population response shown in Fig. 7). This was also the case when we confined our analysis to those neurons that significantly increased their activity according to previous reward omission and/or previous value (*p* = 0.015, Table 1, bottom), indicating conjunctive coding of previous negative outcome and previous positive value as well. Of the 13 AIC neurons concurrently coding previous reward and previous value during the 1-s time window centered around the nose poke entry, nine (69.2%) increased their activity according to previous reward omission and previous value (i.e., negative coefficient for previous reward and positive coefficient for previous value), which is significantly above chance level (25%; binomial test, *p* = 9.9 × 10^−4^). These AIC neurons elevated their discharge rates as a function of previous value, but only when the previous outcome was negative (an example shown in Fig. 7a and population response shown in Fig. 7b). These results show that some AIC neurons conveyed negative prediction error signals in the present behavioral task.

## Discussion

We compared how variables related to uncertain reward are integrated in the rodent AIC and OFC. Although behavioral choice was not needed in the present behavioral task, the results provide new insights on how neural signals related to decision making under risk, namely signals for value, risk and reward, are integrated in the AIC and OFC. Both regions conveyed significant neural signals for all three variables with value and risk signals conjunctively coded. However, value signals were stronger and arose earlier in the OFC, and OFC neurons preferentially responded to the cue predicting the certain reward during cue presentation. On the other hand, AIC neurons tended to elevate their activity following negative outcome, which persisted until the next trial, and according to value in the previous trial. With the caveat that risk-related neural activity might differ depending on the requirement for choice, our results suggest that these differences might underlie distinct contributions of the AIC and OFC to decision making under risk.

### Risk signals in the OFC

A previous study in rats has reported that OFC neurons convey reward probability, but not risk^23^. Also, another study in rats proposed that OFC represents acquired salience rather than risk^25^. However, a significant fraction of OFC neurons modulated their activity according to risk, and their activity was explained better by risk than acquired salience in our study. The reason for this inconsistency is not clear. Both of the previous studies employed odor-cued response tasks with each odor associated with a distinct reward probability, that are similar to our auditory tone-cued response task. Our study is different from the previous studies in that we used five distinct reward probabilities (0, 25, 50, 75 and 100%) instead of four [0, 50, 75 and 100% in ref. 23 and 0, 33, 67 and 100% in ref. 25]. Analysis procedures are also quite different between the previous^23^ and our study, which might have contributed to different conclusions. Although further studies are needed to clarify this matter, our results are consistent with previous studies in monkeys that found significant risk signals in the OFC^19^,^20^, suggesting that risk coding might be a general feature of the mammalian OFC.

### Risk signals in the AIC

Another important aim of the present study was to determine whether and how individual AIC neurons convey risk-related signals. We found that AIC neurons convey risk signals that are distinct from value and reward signals. The magnitude and temporal dynamics of AIC risk signals were overall similar to those of OFC risk signals; they arose at the cue period onset and then declined to chance level within ~1 s after trial outcome was revealed so that they were not maintained until the next trial. Our results indicate that 1) both the AIC and OFC convey significant risk signals and 2) the AIC and OFC concern risk in the on-going trial, but not past trials. However, a difference was found between the AIC and OFC in risk-related neural activity. The majority of OFC neurons responded only to the cue predicting the certain reward during cue presentation, which was not the case for the AIC. Thus, AIC neurons were less biased toward certain reward compared to OFC neurons.

### Conjunctive coding of risk and value

It is well established that subjective value is affected by uncertainty^1^,^2^,^3^. Our finding that the AIC and OFC convey not only value (i.e., objective value which is the reward probability associated with a sensory cue), but also risk signals is consistent with these studies. Moreover, we found conjunctive coding of value and risk signals in both areas; value-coding neurons were significantly more likely to code risk as well than expected by chance. In neurons concurrently coding value and risk, value-dependent activity deviated substantially from a linear function unlike those that coded value only (Fig. 5 compared with Fig. 3b). Such neural activity might reflect subjective value (or utility) that is an outcome of integrating estimated reward probability and risk. These results are consistent with a previous physiological study that found modulation of value-related OFC neural activity by subjective risk attitude of monkeys^20^ (but see ref. 19). Note that our study is limited in that behavioral choice was not involved. It is unknown whether risk-related neural activity in the AIC and OFC is correlated with risk attitude of the rat. It is also unknown whether activity of risk-coding neurons is modulated by reward magnitude. These issues should be addressed in future studies.

### Certain reward-specific response

Our study reveals a novel form of certainty-related neural activity. Some neurons (especially OFC neurons during the cue period) elevated their activity only to the cue predicting certain (100%) reward. In principle, such neural responses can arise by combining risk- and value-related neural signals. For example, a recent study has found neurons that discriminate between cues predicting uncertain (25, 50 and 75%) and certain (0 and 100%) reward delivery in the monkey septal region^26^. Integrating such neural signals with value signals may yield neural responses specific to certain (100%) reward. Our finding of certain reward-specific neurons add to diverse types of linear and nonlinear neural responses to reward uncertainty found in different brain regions, such as the OFC, posterior cingulate cortex, septum and midbrain dopamine neurons^19^,^20^,^26^,^27^,^28^. These diverse neural signals might be used flexibly in reward-based learning and decision making under diverse environmental settings. In particular, existence of certain reward-specific neural responses in the brain structure strongly implicated in computing subjective value (i.e., OFC) raises the possibility that certain reward might be treated specially in computing subjective value. Certainty preference is a well-established characteristics of human decision making^1^. Certain reward-specific neural responses in the OFC might bias the OFC to assign a particularly high value to a certain reward-predicting cue.

Responses of the majority of risk-responsive OFC neurons were better explained by the certain reward model than the base model during the cue period, which was not the case during the response period. This result raises the possibility that reward uncertainty-related OFC signal processing might consist of two steps. The OFC might initially signal whether or not certain (100%) reward is expected along with expected reward value, but does not concern risk quantitatively (quick and imprecise assessment). As the second step, the OFC might convey risk-modulated value signals quantitatively (slow and precise assessment). It will be important in the future to investigate time courses of these OFC processes in reference to behavioral manifestation of the animal’s choice.

### Neural correlates of negative prediction error

Previous studies have found neurons that increase their activity in response to unexpected reward omission in certain subcortical structures such as the habenula, globus pallidus and amygdala^29^,^30^,^31^,^32^. Brain imaging studies also have found activation of several cortical areas, such as the middle temporal gyrus, dorsomedial PFC and AIC, in association with unexpected negative outcomes^33^,^34^. However, neurophysiological studies have not clearly identified cortical areas where neurons preferentially elevate their activity in response to negative prediction error. In our study, previous reward and previous value signals were conjunctively coded in the AIC, and the majority of the AIC neurons concurrently coding previous reward and previous value elevated their discharge rates as a function of previous value, but only when the previous outcome was negative. This result clearly shows that some AIC neurons have a tendency to elevate their activity in response to negative prediction error. Note that a larger number of AIC neurons elevated their activity in response to previous negative outcome without concerning previous value (Table 1). Thus, the AIC conveyed both negative outcome and negative prediction error signals in the present behavioral task. AIC neural activity correlated with negative prediction error might influence the animal to avoid choices that have been associated with disappointing outcomes, thereby contributing to avoiding a risky option with a high expected value.

### Roles of OFC and AIC in risky decision making

Both AIC and OFC conveyed significant value signals that were conjunctively coded with risk signals. These results suggest that both structures might be involved in computing risk-modulated subjective value. However, the OFC conveyed stronger value signals that arose earlier during the nose poke period, suggesting a more important role of the OFC than the AIC in computing subjective value, which is consistent with numerous studies indicating the involvement of the OFC in representing subjective value^35^,^36^,^37^,^38^,^39^,^40^. On the other hand, AIC neurons tended to increase their firing rates following a negative outcome (reward omission) more strongly than OFC neurons, and this tendency persisted until the next trial. These results suggest a more important role of the AIC than the OFC in conveying prolonged negative emotions, which is consistent with previous studies that have shown AIC activation in association with disgust, anxiety and/or disappointment^33^,^41^,^42^,^43^. These signals might influence the animals to avoid choices that have been associated with negative outcomes, especially disappointing ones. In summary, our results suggest the role of the OFC in computing certain reward-biased, risk-modulated subjective value and the role of the AIC in signaling experienced negative outcome and negative prediction error. These OFC and AIC signals might play major roles in determining an individual’s certainty preference and risk attitude.

## Materials and Methods

### Subjects

Three young male Sprague-Dawley rats (approximately 13–14 weeks old, 390–400 g) were water-deprived (maintained >80% of ad libitum body weight) and handled extensively before behavioral training. They were maintained at a 12-h light/dark cycle and performed the behavioral task in the dark phase. All experiments were carried out in accordance with the regulations and approval of the Ethics Review Committee for Animal Experimentation of Ajou University School of Medicine and Korea Advanced Institute of Science and Technology.

### Behavioral task

The animal performed a probabilistic Pavlovian appetitive conditioning task in an open chamber (40 × 40 × 40 cm; black acrylic walls) that had a nose poke hole and a water port on one side (Fig. 1a). Each trial began with the illumination of a blue LED inside the nose poke hole. The animal’s putting its snout into the illuminated nose poke hole (detected by a photobeam sensor) triggered an auditory tone (1 s duration) during which the animal had to maintain nose poke. Nose poke exit following tone offset immediately turned on a blue LED at the water port, and the animal’s arrival at the water port (detected by a photobeam sensor) turned off the LED and triggered water delivery (30 μl) in rewarded trials. Five different auditory cues, that were different in frequency (1.2, 2, 5, 9 and 14 KHz), were associated with five different reward probabilities (0, 25, 50, 75 and 100%) with cue-reward probability combinations varied across rats. The animals had to approach to the water port and break the photobeam in order to proceed to the next trial. Hence, the animals had to respond to all cues including the one predicting 0% reward probability. In trials in which the animals maintained nose poke less than 1 s (incomplete trials), the blue LED at the water port was not turned on and a 5~10-s inter-trial interval was introduced. The next trial began immediately after the outcome was revealed in a complete trial and after the inter-trial interval in an incomplete trial. Each session consisted of a reminder block in which a particular cue was presented continuously for a fixed number of trials (10 trials each for 0 and 100% cues and 30 trials each for 25, 50 and 75% cues; sequence of cues randomized; total 110 trials) and a test block in which the five cues were presented in a random order for 200 trials. The task progressed to a new, randomly determined trial type following both complete and incomplete trials in the test block so that the animals were unable to predict the identity of the next cue in all trials. Only the neural data collected during the test block was analyzed.

### Neurophysiological recording

A microdrive array containing 12 tetrodes (diameter of the tetrode bundle: ~1 mm) was implanted on the left (n = 2) or right (n = 1) side of the brain targetting the lateral OFC and AIC [3.2 mm anterior and 3.2 mm lateral to bregma with a two degree angle away from the midline under deep anesthesia with sodium pentobarbital (50 mg/Kg; i.p. injection)]. The tetrodes were gradually lowered into the intended recording areas over 16–19 days. Once the recording began, all tetrodes were advanced by 75~150 μm after a daily recording session. Unit signals were amplified 3000~10000×, band-pass filtered between 0.6–6 KHz, digitized at 32 KHz and stored on a personal computer using the Cheetah data acquisition system (Neuralynx; Bozemann, MT, USA). When recordings were completed, small marking lesions were made by applying an electrolytic current (30 μA, 20 s, cathodal) through one channel of each tetrode. The anatomical location of each recorded unit was determined based on the location of a marking lesion (histological examination of 40 μm coronal brain sections under a light microscope) and the advancement history of the corresponding tetrode^44^ (Fig. 1c,d).

Putative single units were isolated off-line by examining various two-dimensional projections of spike waveform parameters using MClust software (A.D. Redish). Only those clusters with no inter-spike interval <2 ms, L-ratio <0.2 (0.03 ± 0.02, mean ± SD) and isolation distance >12 (48.34 ± 47.30, mean ± SD)^45^, were included in the analysis. The recorded units were classified into putative pyramidal cells (spike widths ≥ 0.22 ms) and putative interneurons (spike widths <0.22 ms). The mean (±SEM) firing rates of putative pyramidal cells and interneurons were 3.22 ± 0.29 and 7.84 ± 1.87 Hz, respectively, in the AIC, and 3.04 ± 0.25 and 8.08 ± 1.70 Hz, respectively, in the OFC.

### Multiple regression analysis

Neural signals related to value, risk and reward were estimated using the following regression model (base model):


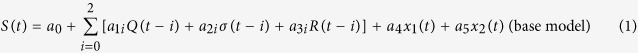


where *S*(*t*) indicates spike discharge rate in trial *t*, *Q*(*t*) is expected value (i.e., reward probability; 0, 0.25, 0.5, 0.75 and 1 in 0, 25, 50, 75 and 100% reward probability trials, respectively), σ(*t*) is risk (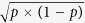, where *p* is the reward probability; 0, 0.433, 0.5, 0.433 and 0 in 0, 25, 50, 75 and 100% reward probability trials, respectively), *R*(*t*) is reward (i.e., trial outcome; 1 and −1 in rewarded and unrewarded trials, respectively), *i* is trial lag (0, current trial; 1, previous trial; 2, two trials before), *x*_1_(*t*) and *x*_2_(*t*) are nose poke duration and reaction time (the time interval between nose poke exit and water-port entry), respectively, and *a*_0_~*a*_5_ are regression coefficients.

To distinguish responses specific to the cue predicting the certain reward (100%) from other types of risk response, the base model (eq. 1) was compared with the following model (certain reward model):


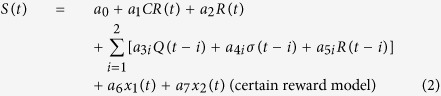


where *CR*(*t*) denotes certain reward-specific response (1 for 100% reward probability and 0 for all other probabilities).

We also tested whether activity of a given neuron is better explained by risk or ‘acquired salience’^24^,^25^. Acquired salience is determined by the weighted sum of conditioned stimulus-unconditioned stimulus (CS-US) and CS-absence of the US (CS-noUS) associations^24^. To test whether neuronal activity is better accounted for by acquired salience or risk, the following two models were compared:


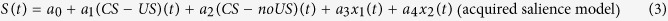



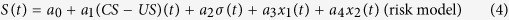


where (*CS* − *US*)(*t*) is the association strength between a CS and US in trial *t*, which is equivalent to expected value [*Q*(*t*)] in eq. 1, and (*CS* − *noUS*)(*t*) is the association strength between CS and noUS (i.e., no reward delivery) (0, 0.75, 0.5, 0.25 and 0 in 0, 25, 50, 75 and 100% reward probability trials, respectively).

### Statistical tests

Statistical significance of a regression coefficient was determined with a *t*-test. Significance of the fraction of neurons for a given variable was determined using a binomial test. Significance of the difference in the fraction between activity-increasing vs. -decreasing neurons and between AIC and OFC neurons was determined with a χ^2^-test. However, Fisher’s exact test was used instead of a χ^2^-test whenever the expected number is less than five. A *p* value <0.05 was used as the criterion for a significant statistical difference. All data are expressed as mean ± SEM unless noted otherwise.

## Additional Information

**How to cite this article**: Jo, S. and Jung, M. W. Differential coding of uncertain reward in rat insular and orbitofrontal cortex. *Sci. Rep*. **6**, 24085; doi: 10.1038/srep24085 (2016).

## Supplementary Material

Supplementary Information

## Figures and Tables

**Table 1:** Conjunctive coding of two different variables.

Region	Period	*Q*(*t*)	*σ*(*t*)	*Q*(*t*) & *σ*(*t*)	*p* value
AIC	Cue	19	14	8	^F^2.1 × 10^−5^
	Response	39	20	10	0.006
	Reward	26	20	8	^F^0.007
OFC	Cue	47	27	10	0.072
	Response	45	27	15	8.8 × 10^−6^
	Reward	37	21	9	^F^0.005
AIC	−0.5s ~ 0.5s from trial onset	*Q*(*t* − 1)	*R*(*t* − 1)	*Q*(*t* − 1) & *Q*(*t* − 1)	*p* value
		18	75	13	0.029
		(+)*Q*(*t* − 1)	(−)*R*(*t* − 1)	(+)*Q*(*t* − 1) & (−)*R*(*t* − 1)	*p* value
		15	47	9	^F^0.015

Conjunctive coding was tested by examining numbers of neurons coding each and both variables. *P*-values were determined with a χ^2^-test or Fisher’s exact test when the expected number is less than five (indicated by ^F^). (Top) Conjunctive coding between current value [*Q*(*t*)] and current risk [σ(*t*)] signals during the cue, response and reward periods (1 s each) in the AIC and OFC. (Bottom) Upper, conjunctive coding between the previous value [*Q*(*t* − 1)] and previous reward [*R*(*t* − 1)] signals in the AIC during the 1-s time period centered around cue period onset. Lower, Conjunctive coding between the positive previous value [(+)*Q*(*t* − 1)] and negative previous reward [(−)*R*(*t* − 1)] signals in the AIC during the same time period.
