# Supplementary material for: Differential coding of uncertain reward in rat insular and orbitofrontal cortex
Source: Sci Rep. 2016 Apr 7;6:24085. doi: 10.1038/srep24085 (PMC4823699; doi:10.1038/srep24085)
Supplement: Supplementary Information [file srep24085-s1.pdf]

Distinct roles of insular and orbitofrontal cortex in anticipating and evaluating uncertain reward

Suhyun Jo[1,3] and Min Whan Jung[1,2,3]

Supplementary figures and table

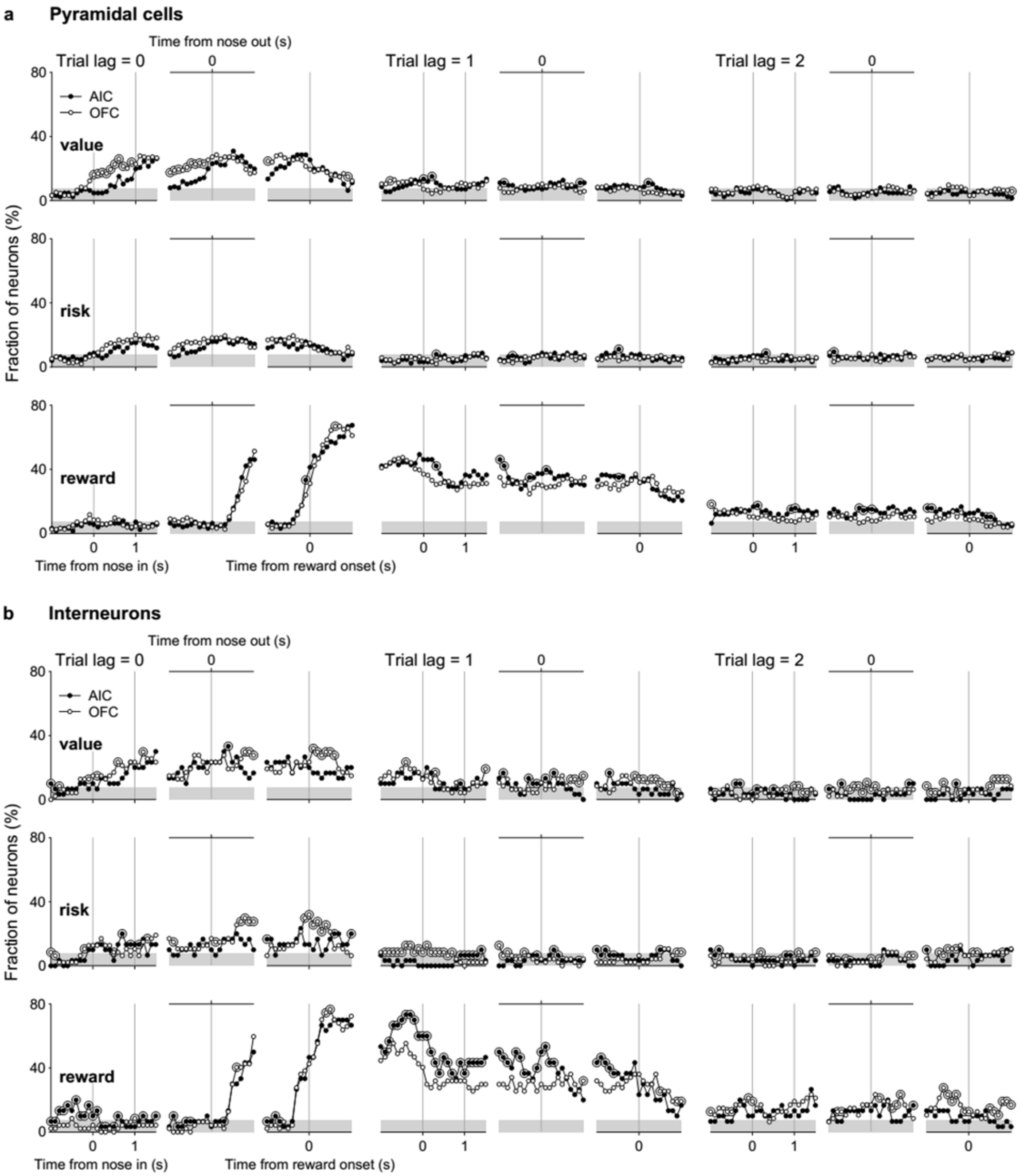

Figure S1. Similar results were obtained regardless whether all units or only putative pyramidal cells were analyzed. Shown are temporal profiles of neural signals for value, risk and reward plotted separately for putative pyramidal cells (**a**) and putative interneurons (**b**). Time courses of neural signals for value, risk and reward were similar between putative pyramidal cells and interneurons, possibly because inhibitory interneurons are excited by a large number of surrounding pyramidal cells.

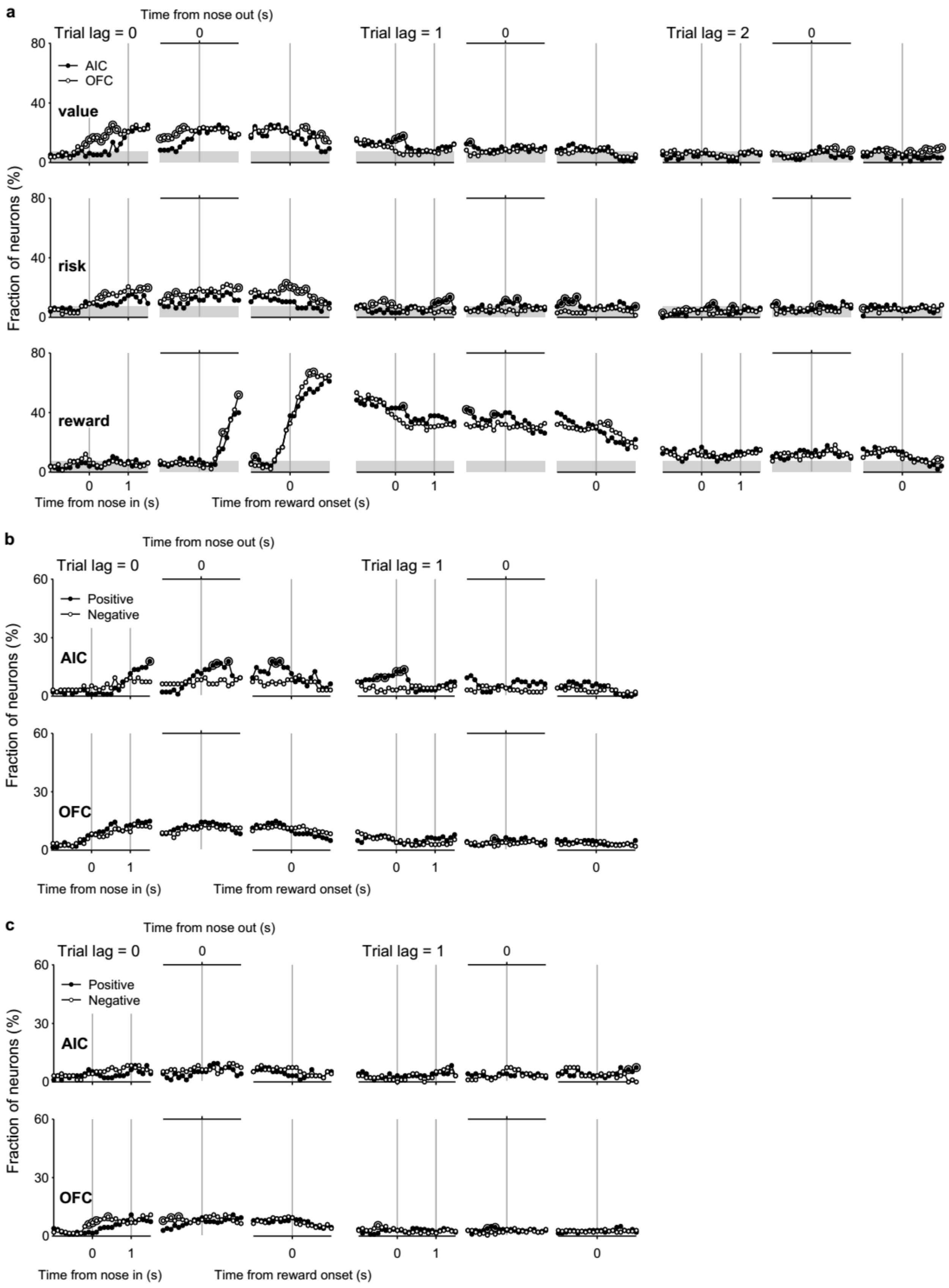

a
Time from nose out (s)
Trial lag = 0
Trial lag = 1
Trial lag = 2
AIC
OFC
value
risk
reward
Fraction of neurons (%)
Time from nose in (s)
Time from reward onset (s)
b
Positive
Negative
AIC
OFC
c

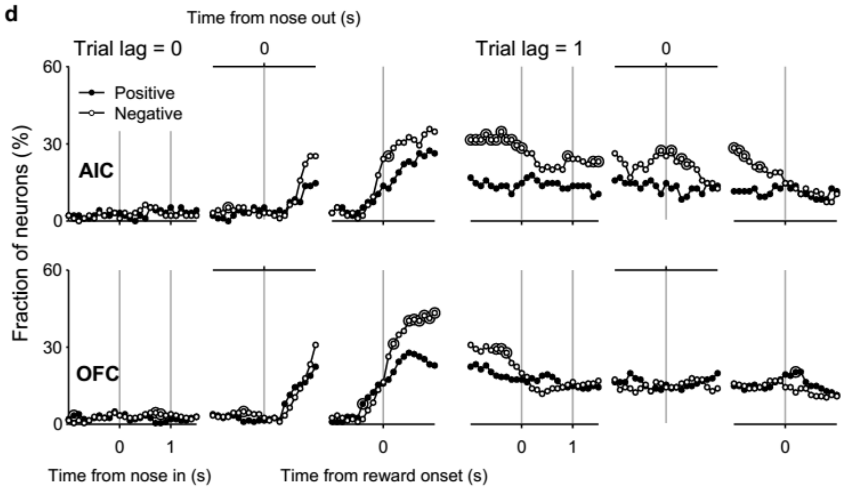

Figure S2. Similar results were obtained when those neurons located near the border between the AIC (n=61 out of 156) and OFC (n=70 out of 201) were excluded from the analysis. (**a**) Fractions of neurons for value, risk, and reward in the AIC and OFC. Same format as in Figure 2d. (**b-d**) Positive (activity-increasing) and negative (activity-decreasing) types of value-, risk-, and reward-responsive neurons. Same format as in Figure 3a, 4a, and 6a, respectively.

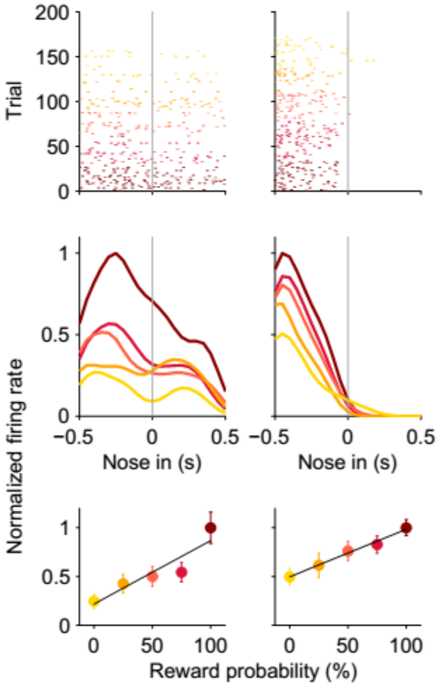

Figure S3. Example AIC neurons significantly responding to previous value (reward probability in the previous trial) around cue period onset (time 0). Same format as in Figure 2a.

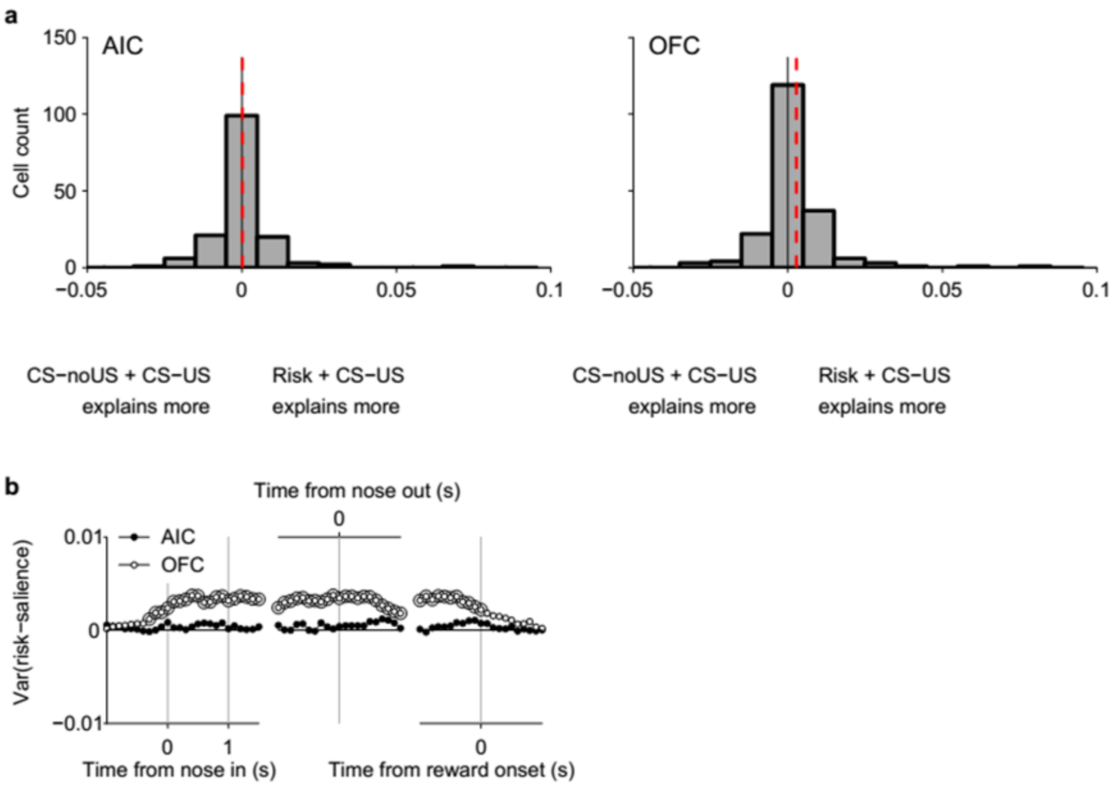

Figure S4. Comparison between the risk and acquired salience models. **a**, Analysis results for the cue period. The frequency histograms show distributions of the difference in variance explained by the risk and acquired salience models (variance explained by risk model – variance explained by acquired salience model) for all analyzed neurons (AIC, n=156; OFC, n=201). The mean value (red dashed line) was significantly larger than 0 (i.e., neural activity was better accounted for by the risk than acquired salience model) for the OFC. **b**, Results from a sliding window (1-s duration advanced in 0.1-s time steps) analysis. The ordinate is the mean difference in variance explained by the risk and acquired salience models (corresponding to the red dashed line in A). Large open circles indicate significant deviation from 0 (two-tailed *t*-test).

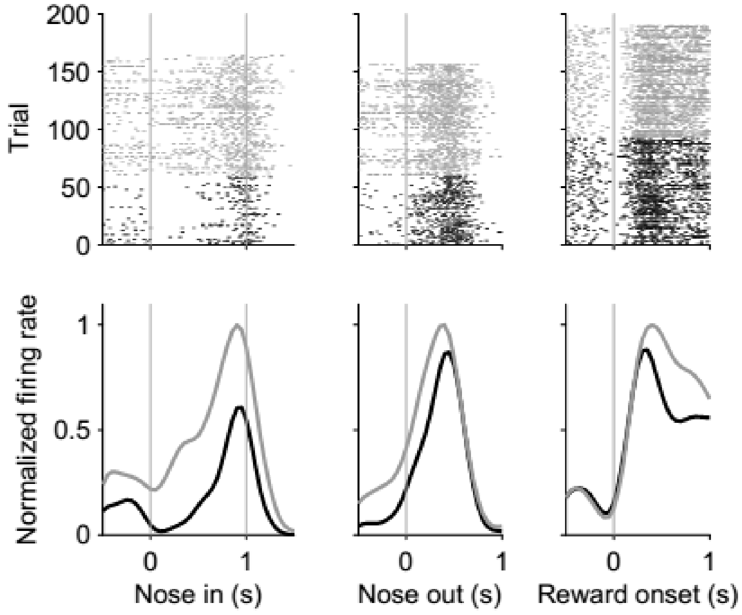

Figure S5. Example AIC neurons significantly responding to previous reward (black, rewarded; gray, not rewarded) during the cue (left), response (middle), or reward period (right). Same format as in Figure 2a.

Table S1. Fractions of neurons responsive to value, risk, and reward. Neural data during the cue and response periods was analyzed for value- and risk-related neural activity, and that during the first 1 s of the reward period was analyzed for reward-related neural activity. Statistical significance (*p*-value) was evaluated using the binomial test as well as a permutation test. For the latter, trials of a given variable were randomly shuffled (with trials of spike data and all other independent variables of the multiple regression model remained fixed), the regression coefficient for the variable was obtained, and the fraction of neurons responsive to the variable was counted. This procedure was repeated 1,000 times, and the *p*-value was determined by the frequency in which the fraction of neurons obtained after trial shuffling exceeded the original fraction of neurons.

Region	Coding	Period	No.	Binomial	Permutation
AIC	Value	Cue	19	$3.2\text{x}10^{-4}$	0
		Response	39	$4.9\text{x}10^{-17}$	0
	Risk	Cue	14	0.025	0.005
		Response	20	$1.1\text{x}10^{-4}$	0
	Reward	First 1 s of reward	92	$1.0\text{x}10^{-35}$	0
OFC	Value	Cue	47	$6.3\text{x}10^{-19}$	0
		Response	45	$2.1\text{x}10^{-17}$	0
	Risk	Cue	27	$3.3\text{x}10^{-6}$	0
		Response	27	$3.3\text{x}10^{-6}$	0
	Reward	First 1 s of reward	135	$1.0\text{x}10^{-35}$	0
